# Repertory of the Back

**Published:** 1897-12

**Authors:** E. H. Wilsey

**Affiliations:** Parkersburg, W. Va.


### REPERTORY OF THE BACK.

Dr. E. H. Wilsey, Parkersburg, W. Va.

(Continued from page 448.)

**Chill** from back to arms, Calc-ars.

— with violent fever, especially in back he cannot get warm, and yet has internal heat, Nit-ac.

**Chilliness** in back in general, Ang., *Camph.*, *Cham.*, Crot-t., *Dig.*, *Dulc.*, *Ham.*, Lept., *Nat-m.*, Nitr-sp-d., *Puls.*, *Raph.*, Sec., Spong., Guaic., Stram., Benz-a., *Lyc.*, *Gels.*, *Cepa.*, Sil., *Eup-pur.*, *Con.*, *Sul.*, *Coff.*, *Nux-v.*, *Stront.*, *Aesc-h.*, *Kali-b.*, *Cap.*, Lachn., Ascl-s., Merc-s., Aph-ch., Pic-ac., Alum.

— in back with cold feet, *Lyc.*

— in draught of cold air, Dulc.

— down back when changing position ever so little, *Eup-pur.*

— down back, Bry., *Cepa.*, *Gels.*, Sil., Ceanothus.

— down back in region of spine during damp weather, Pic-ac.

— in back after eating, Sil.

— in back in evening, *Dulc.*

— in back in evening after severe cold, Sep., *Sul.*

— in back in evening with dry heat after lying down, *Coff.*

— in back after lying down in evening, *Nux-v.*

— in back when pains there begin to abate, Asclep.

— pains in back before and after chilliness, Diad.

— pain in back with chilliness, icy cold feet and stranguary, Elaps.

— from suppressed menses with pain in back, *Puls.*

— of back with heat in face and head, Jatroph., *Sul.*, Nitr-sp-d.

— in back with emaciation, redness of cheeks and insomnia, Hepar.

— in back during influenza, in morning as if skin had been frozen, *Nux-v.*

**Chilliness** in back, seeks a warm place, *Kali-b.*
— in back, worse at night, *Stront.*
— in back like cold water poured down, *Puls.*, Alum., Stram., Zinc.
— in back, as if cold water was dropping down, during chill in intermittent fever, *Caps.*
— as if cold water was spurted upon back at 6 P. M., *Lyc.*
— frequent on arms, back, thighs, and abdomen, Sarsa.
— in back in P. M., Guiac.
— running up and down back, Aphis.
— a glow on back after chilliness, Ars-hyd.
— and stiffness at nape of neck and all down back, Sil.
— constant in back chiefly, Dig.
— in back and posterior surface of arms, Raph.
— in back, as if sweat would break out, Camph.
— on back, without thrist, in open air, but especially in a draft, Dulc.
— creeping in back from above downward, Bry.
**Chills** over back and limbs, Agari.
— creeping on back principally, *Eup-per.*
— in back, *Tromb.*
— intense fever commencing with chills running down back like streams of cold water, causing shivering and chattering of teeth, Variol.
— distressing chills in lower part of back, calves of legs cold, beginning in spine just above sacrum, Ox-ac.
— begin in lower dorsal region and run up back, *Eup-pur.*
— and creeps running up back, *Gels.*
— internal chill for several afternoons, for a half or a full hour, at times a sensation as of hot water in the scorbiculus cordis and in back, Phos.
— could not sleep during whole menstruation on account of tearing in back, chills and heat with thirst and painful contractions of chest, *Sep.*
— run up back, Cepa.

**Chilly** sensation on shoulders and down back, Leptand.
— cold feeling in small of back, *Dulc.*
— sensation along back like cold water, Stram.
— strong aversion to air, if air is let under bedclothes while turning over it makes him chilly, *Nux-v.*
— crawls run down back most at night, with more frequent urination followed by heat and thirst, Cepa.

**Circular,** back bent often in circular form, Opi.

**Clawing,** pain in small of back like a clawing, sticking, drawing, and working all together, Ign.

**Clucking** strong motion in muscles of left side of back only when lying on right side, Ars.

**Clutching,** several paroxysms daily of half-hour duration, first of griping and clutching in back, whence it comes like a shooting into side; things become black before her eyes and she must lie down wherever she is, Lyc.

**Coition,** burning pain in back mornings, waking him after evening coition, worse from rest and better by motion, *Mag-m.*

**Coffee,** backache worse from coffee, stooping, and walking, *Cham.*

**Cold,** heat in evening with a thrill of cold and a shudder over the back without thirst, Nat-m.
— pain in small of back after heavy lifting and taking cold at same time, *Sul.*
— lameness in small of back from cold, Dulc.
— back painful after taking cold, *Dulc.*
— constant pain in back and hips with cold limbs, Pallad.
— pain in back after taking slightest cold, *Nit-ac.*
— liable to catch cold causing pain in back, Nit-ac.
— icy in back and between scapula a spot where formerly a pain had been only internally and not to be warmed by feathers or wool; after half a day cold turned into an itching, Amm-m.
— creeps up back evening and night, *Ars.*

**Cold,** sensation in back, Croc.
— stiffness, as after taking cold in back and sides, Sul.
— trickling down on both sides of upper arm, over back and feet while yawning, Mez.
— flushes of heat or currents of cold air down back, Cof-cr.
— back is cold while there is a dry heat in thighs and sacrum, *Sul.*
— numbness, pricking causing a cold sensation, Ox-ac.
— aching in small of back as after taking cold, Ars-hyd.
— drawing in back, upper and lower limbs and fingers, as after taking a cold, Dig.
— first some chilliness down back with icy cold hands, then intense heat with distention of abdomen, Sil.
— feeling on back, Camph.
— severe rheumatic stitches in back and arms after taking cold while sweating, worse at night and while resting, better from motion; slight fever, much thirst, *Dulc.*

**Cold Water,** intense fever commencing with chills running down back like streams of cold water, Variol.
— chilly feeling along back like cold water, Stram.
— sensation like cold water poured down back, *Puls.*
— sensation as if cold water was dropping down back, *Cap.*

**Coldness,** of back in general, Arum-t., *Ars.*, *Amm-m.*, Brach., *Cact.*, *Calc.*, Croc., *Camph.*, Elaps., Cof-cr., *Lac-def.*, *Opi.*, Lact-v., Mur-ac., *Nat-m.*, Mez., Kali-c., Rhus, Sec., Sul., *Sep.*, *Ver.*
— in small of back and loins, *Camph.*, worse by walking a few steps.
— in evening at 7 o'clock a shaking ague and great coldness, as if she lay on ice for two hours, with drawing pains in all limbs and back, and on waking from a sleep full of dreams, sweat all over and severe thirst after the sweat, Lyc.
— violent pain in small of back with great coldness, Amm-c.
— and feeling of numbness on the side of back on which he lay during his siesta, Calc-c.

**Colic,** pressing, crampy labor-like colic in small of back and bladder, C-veg.

**Confinement,** backache after confinement, *Hyper.*

**Confusion in back,** extending through nape into head, Iod.

**Consciousness,** during short intervals of consciousness complains of great sensitiveness of back, neck, and whole spine, *Coccul.*

**Constant** pain night and day in back, Zinc.

— pain along back, worse in those parts she had to lie on, *Fer.*

— pain in small of back, *Lac-def.*

**Constipation** with backache, Psor.

— with pain in small of back, *Lach.*

**Constriction** painful in back, *Graph.*

— in back, *Cetrar.*

— and pain in muscles of back, *Dulc.*

— tensive, extending down to small of back, Mez.

**Contracted,** muscles of back contracted, drawing head backward, Ver-v.

**Contractive,** spasmodic pain in small of back, *Mag-m.*

— pain in stomach, with sensation of coldness in it and in the back awaking her from sleep in the morning, Con.

— pain in back, with contractive sensation between scapula, Guaic.

— pain in back after physical labor, while at rest, Kali-c.

— pain in stomach, extending into back after stool with burning in anus, Mag-m.

— pain violent in both shoulders, and tearing down back in morning, Mag-c.

— pain in back, *Graph.*

**Coryza,** aching in small of back as if bruised, with coryza, Cinnab.

— severe stuffed coryza all the day with pain in back, especially while sitting, Zinc.

**Cough,** during the cough sensation as if the lungs touched the back, Sul.

— burning heat in left chest through to back with cough, Oleum-jec.

— pressing pain in small of back with whooping cough, Spong.

— during cough pain in back, Nitrum.

— pain in back striking through to sternum with cough and expectoration of tough black mucus, *Kali-bich.*

**Coughing,** stitches in small of back when coughing, breathing deeply, or walking, Arn.

— pain in back on coughing, ascending stairs, stepping hard, and jumping, preventing deep breathing, and causing her to have a fever, Carbo-a.

— sticking in back, extending toward chest when coughing, Psor.

— stitches in back when coughing, *Sepia.*

— aching in small of back, worse by either coughing or laughing, Tell.

**Cracked,** rheumatic pains in back while stooping, felt as if something cracked across sacrum, cannot stoop or move for pain, which remains constant, even while at rest, but worse on least movement of trunk or legs, *Kali-bich.*

**Cracking** sound from first cervical vertebræ; on rising from sitting, with stiffness in left side of neck, Zing.

— in small of back while walking, Zinc.

**Cramp** in back, Lyss., Hydroc-ac.

— in back in brain affections, *Hell.*

— in back in hypochondriasis, Arg-n.

— in back and in ribs in front with pearls of sweat in the face and on the arms for three-fourths of an hour, then a profuse mucous diarrhœa, Petrol.

— in back just below hips in morning, Alum.

— in back after menses, Bor.

— insupportable pain in small of back like cramp, worse from least motion, *China.*

**Cramp** like pain in back, Euphorb., Euphr.

— like severe pains in small of back with cramp-like drawing in umbilical region, *Cof-tosta.*

— like constriction or pulsation ascending from thighs into small of back, Ruta.

— like pressive pain in back in renal region, Caust.

**Cramps,** violent in back and in feet, Iod.

— after menses in evening, coldness in back and awaking after midnight with cramp and coldness of stomach, which continues till about noon, Kali-c.

— in muscles of back and abdomen on turning head and body, Dros.

— like pains in back and legs, *Arg-n.*

**Crawling** in back, Agari., Ars-h., Ars., Berb., Calend., *Cepa., Cham.,* Asa., Caust., Sec., Ars-m., *Lyc., Gels., Sul.,* Variol., Ang., *Crotal.,* Pallad., Osmium.

— on back as from ants, Graph.

— in back as from fleas, Pallad.

— over shoulders and back, Ars-h.

— itching on back and shoulders, as from crawling insects, worse evening on going to bed, Osmium.

**Crawls** run over back in P. M., Asaf.

— chilly crawls running down back most at night, with more frequent urination, followed by heat and thirst, Cepa.

**Creeping,** sensation of hot water creeping through back from below upward, Nitri-s-d.

— a gentle creeping sensation in back, as if a soft air were blowing through it, Sec.

**Crick** in neck or back, Fer-ph., Calc-ph., Agari.

— in back of eight years' standing, Sepia.

— in back caused by a strain, Sep.

— in back, better by continual motion, Sep.

— in back, worse when first beginning to move, Sep.

— in back, a jar or a misstep hurts her back severely, as also riding, Sep.

— when walking in open air a pain darted through into small of back, "crick in back," most painful on rising after long-continued sitting, Amm-c.

**Cries,** excruciating, tearing pains in back, extorting cries, cause him to bend up during chill, *Cap.*

**Crookedly,** painful stiffness in small of back which compels him to sit and walk crookedly, Bry.

— feeling as if middle of back were crooked, Raph.

**Crushed,** pain in small of back, as if bruised or crushed in rest and in motion, also at night in bed, so she cannot lie on back nor on either side, *Amm-m.*

**Curvature** of dorsal vertebræ, *Calc-c.*

**Curved,** bent backward spasmodically like an arch, violent trembling motion, *Opium.*

**Cut,** pain in back, as if cut in two, can neither walk, stand, nor lie, Hep.

**Cutting** in back in general, *Arg-n.*, Aur-m., *Calc-ph.*, Eup-pur., *Gels.*, Zinc., Guarea, *Nat-m.*, *Coloc.*, Canth., Mag-m., Rhus-t., Cup., Sil.

— violent cutting pain in back, Eup-pur.

— sharp cutting pain from back down hips, *Gels.*

— pain about the lower dorsal vertebræ and aching in back, *Asclep.*

— burning in back, with cutting and tensive pain, Aur-m.

— pain in back on left side, in region of lumbar vertebræ, a drawing cutting, diminished by pressing on it by the hand, Dig.

— in a small strip of left side of abdomen over against the back, then rolling in the abdomen while the pain goes off, Sarsa.

— painful in back all day long, Sil.

— violent cutting in small of back from slightest movement, extending into calves and feet, so that he can neither stand, walk, nor lie, Zinc.

— in back and abdomen, extending through urethra, Canth.

— and sticking through back and abdomen, Canth.

**Cutting** pulsating feeling in back, *Nat-m.*
— and pinching below the navel with shivers over the back, then heat in back and urging to stool at noon, Mag-m.
— pressure, as with a cutting edge across small of back while standing or bending backward, Rhus-t.
— sharp cutting drawing in left side of back, Cup.
— in small of back and groin, *Arg-n.*
— in back all day, Sil.
— in small of back during too early menses, *Ol-an.*
**Dark,** pains in back, cannot walk with eyes closed in dark, *Arg-n.*
**Day,** violent pain in back all day, Camph.
— sudden pains in small of back in morning and the whole day, not at night, Ant-cr.
**Darts** in small of back, *Kali-iod.*
**Darting** across right side of small of back, stooping, passing away gradually, Lact-ac.
**Deglutition,** painful jerk in back at every deglutition, also during abortive eructation, sometimes without deglutition, when at rest; but every time afterward there follows an arrest of breathing, Petrol.
**Deliriously**, if he lies at night on his back or on his right side, he starts up and talks deliriously, and cries out about his frightful dreams, Mag-c.
**Denuded** sore sensation in left side of back when sitting, with burning intermittent sticking, Plat.
**Diarrhœa,** soreness in small of back and rectum, as with diarrhœa, Jat.
**Dinner,** bruised pain in back when sitting an hour and one-half after dinner, better by walking, Phell.
— about an hour after dinner (even before) a dragging pain in stomach and a gnawing extending to back, where it is most acute, then great exhaustion and lassitude, Sep.
**Diphtheria** with backache, *Lyc.*, *Phyto.*, *Lach.*

**Dislocated,** pain in cervical vertebræ, as if dislocated when lifting arms, *Ang.*

— pain in left side of back, as from dislocation, extending to left hypochondrium, Lyc.

**Diseases** brought on by exposure of back to draft of air, *Sil.*

**Distress,** aching distress in small of back, *Lac-def.*, Ustil.

**Dorsal,** a chill begins in lower dorsal region and runs up back, *Eup-pur.*

— pain in upper dorsal vertebræ, Aletris., Stram.

— pain constant in cervical and upper dorsal vertebræ, Stram.

— pain in right side of back between tenth dorsal vertebræ and side, Benz-a.

— violent pain in top of right shoulder and shortly afterward in back from about first to fourth dorsal vertebræ, *Lob-i.*

**Down,** pain in back low down, Lappa.

— pain from small of back downward, *Nux-m.*

**Dragging** pain in back in morning on waking, Myrica.

— pain in small of back, *Con.*

— in back as before menses, followed by slight show, Canth.

**Drawing** pain lasting for several hours, starting from the back, seemingly in the middle of the chest in the œsophagus, in P. M., Agari.

— in back as if bruised, thence the pains extend to sacrum and the abdomen, where much flatus accumulated with bellyache, and as the flatus was discharged, leucorrhœa appeared, Caustic.

— pain in small of back, Cycla., Tereb., *Kali-c.*, *Nit-ac.*

— pain in back in evening, Carbo-v., Nit-ac.

— pain in the entire back, followed by fatigue, *Card-mar.*

— pain in back in A. M., Ars.

— pain in small of back, as if heavy body was moving downward, Bar-c.

— pain in back, Valer.

**Drawing** pain in post-hepatic region, extending through to back, *Calc-c.*

— pain in back, also gastric region, Hepar.

— pain in back on left side in region of lumbar vertebræ, a drawing cutting, diminished by pressing on it with the hand, Dig.

— pain from small of back into legs, Amm-c.

— suppression of menses which had just appeared, when there followed by day and by night a drawing pain down back, Con.

— pain in back with tense feeling as before menstruation, Mosch.

— pain in back on moving and treading, Sul-a.

— stinging or tearing pain in back, *Cham.*

— painful in back and limbs, *Chel.*

— pains from small of back to shoulders, *Ars.*, Dros.

— pain from small of back down thighs during rest; stitches when moving, better by pressure, *Dulc.*

— tearing pain in back and spine, *Cap.*

— pain in back when sitting, Thuja, Carbo-v.

— pain in small of back and a feeling as if it was broken, when walking, standing, and lying, *Carbo-a.*

— pain in sacrum and back, also a sensation of going to sleep in shoulder joint, disturbing sleep and disappearing on waking, Zinc.

— pain in right side of back from shoulder-blade to lumbar region, changing locality every minute, *Card-m.*

— pain in small of back, sacrum, coccyx, and thighs, better by walking, and worse on turning trunk while standing, Thuja.

— pain in back extending upward, Nat-m.

— at night drawing pain in back, she had to turn over frequently to get relief, Nat-m.

— in back in general, Ast-r., *Cham.*, *Chin.*, Crotal., Lyss., *Nux-v.*, Op., Zing., Calc-ph., *Lil-tig.*, *Merc.*, *Lach.*, *Coc-c.*,

Lyc., Nat-c., *Kreos.*, Con., Bry., *Colch.*, Staph., Carbo-v., Ter., *Sil.*, Ars., Graph., *Carbo-a.*, Hep., *Chel.*, *Ant-t.*, Sang., *Cap.*, *Carduus*, *Puls.*, Ind., Calab., Ver-v., *Nat-m.*, *Sabin.*, Petrol., Nit-a., Sul-a., Sul., Stram.

**Drawing,** dull aching pain in back between shoulders at 6 P. M., Zing.

— drawing pain in small of back extending into pubic region, *Sabina.*

— burning along back, beginning at coccyx, as if under skin, Mur-a.

— on back, ceasing on bending backward, Petrol.

— burning in small of back, Zinc.

— in back, upper and lower limbs and the fingers, as after a cold, Dig.

— in all limbs and back in the evening at 8 o'clock, for two hours, and great coldness even in bed, Lyc.

— disabling in small of back, *Coccul.*

— in lower part of back, as from incarceration of flatus, Nat-c.

— in back and limbs with gaping, Calc-ph.

— spasmodic from behind forward, into genitals or down into thighs, *Kreos.*

— intolerable in small of back and down into legs, evening, *Lach.*

— sharp in back and in through hips, *Nat-m.*

— down back with heaviness of limbs, Ant-t., Carbo-v.

— lacerating in back, Opi.

— upward from foot extending into back when moving, Nit-ac.

— spasm in back with severe pressing and drawing, Con.

— a drawing down back when sitting, disappearing when in motion, Bry.

— pressure in first two dorsal vertebræ with soreness, Staph.

— in back on motion and on stooping, Sul-a.

— tension and stitches occurring in back, much worse on motion, *Colch.*

**Drawing** in back while sitting in evening, Tereb.
— in back mostly when sitting, Carbo-v.
— a spasmodic drawing in back, compelling him to lie still, *Sil.*
— up and down back, Ars.
— extending from small of back up, *Lach.*
— violent drawing in back, Graph.
— sharp across back, sensitive to every step, *Carbo-a.*
— pressure in back, Kali-c.
— stitches in small of back with drawing through lumbar vertebræ while standing, *Con.*
**Drawn,** the back is drawn backward, Stram.
**Driving,** aching in back when driving in evening, Sul.
— tensive pressure like severe fatigue from right scapula, extending into side of back even to sacrum, also early in bed, but especially while driving, Kali-c.
**Dreams** that his back is covered with warts and excrescences, Mez.
— in his dreams at night he always lies on his back, Mang.
— anxious dreams with sweat on back when waking, Hepar.
**Dull** pain in small of back with drowsiness and lassitude, Cornus.
— pain across small of back, Cund.
**Dysmenorrhœa** with pain in small of back, *Lach.*
**Dyspnœa** with pain in small of back, *Lach.*
— sensation of weight in back and shoulders with dyspnœa, *Nat-m.*
**Eating,** backache better by lying down and by eating, *Nat-m.*
—pain in back after eating, *Kali-c.*
— anxiety and uneasiness in back after eating, Carbo-a.
— pain in back as from fatigue, especially after eating, and while sitting, Ant-t.
— fullness, anxiety, and tearing pain in back after eating; afterward extending into abdomen, Cham.

